# Transcriptome-Based Identification of Candidate Flowering-Associated Genes of Blueberry in a Plant Factory with Artificial Lighting (PFAL) under Short-Day-Length Conditions

**DOI:** 10.3390/ijms25063197

**Published:** 2024-03-11

**Authors:** Haishan An, Jiaying Zhang, Shuigen Li, Xueying Zhang

**Affiliations:** 1 Forestry and Fruit Tree Research Institute, Shanghai Academy of Agricultural Sciences, No. 1000 Jinqi Road, Fengxian District, Shanghai 201403, China; anhaishan@saas.sh.cn (H.A.);; 2 Shanghai Key Lab of Protected Horticultural Technology, No. 1000 Jinqi Road, Fengxian District, Shanghai 201403, China

**Keywords:** *Vaccinium corymbosum* L., plant factory, candidate flowering genes, chilling

## Abstract

In blueberry (*Vaccinium corymbosum* L.), a perennial shrub, flower bud initiation is mediated by a short-day (SD) photoperiod and buds bloom once the chilling requirement is satisfied. A plant factory with artificial lighting (PFAL) is a planting system that can provide a stable and highly efficient growing environment for blueberry production. However, the characteristics of bud differentiation of blueberry plants inside PFAL systems are poorly understood. To better understand flower bud initiation and the flowering mechanism of blueberry in PFAL systems, the anatomical structure of apical buds under SD conditions in a PFAL system was observed using the southern highbush cultivar ‘Misty’ and a transcriptomic analysis was performed to identify the candidate flowering genes. The results indicated that the apical bud of ‘Misty’ differentiated gradually along with SD time course and swelled obviously when chilling was introduced. A total of 39.28 Gb clean data were generated, and about 20.31–24.11 Mb high-quality clean reads were assembled, yielding a total of 17370 differentially expressed genes (DEGs), of which 9637 were up-regulated and 7733 were down-regulated. Based on the functional annotation, 26 DEGs were identified including 20 flowering-related and 6 low-temperature DEGs, out of which the expressive level of four flowering-related DEGs (*VcFT2*, *VcFPA*, *VcFMADS1*, and *VcCOP1*) and two low-temperature-induced DEGs (*VcTIL-1* and *VcLTI 65*-like) were confirmed by qRT-PCR with a good consistency with the pattern of transcriptome. Functional analysis indicated that *VcFT2* was highly conserved with nuclear and cytoplasmic subcellular localization and was expressed mainly in blueberry leaf tissue. In *Arabidopsis*, ectopic overexpression of *VcFT2* results in an early flowering phenotype, indicating that *VcFT2* is a vital regulator of the SD-mediated flowering pathway in blueberry. These results contribute to the investigation of photoperiod-mediated flowering mechanisms of blueberry in PFAL systems.

## 1. Introduction

Blueberry (Ericaceae, *Vaccinium* spp.) is an economically important small woody perennial bush that includes several ecotypes, such as lowbush, semi-highbush, highbush, and rabbiteye blueberries, and interspecific hybrids, and the cultivation area of blueberry in China has statistically achieved about 66,400 ha with an annual yield of 0.35 million tons [1,2]. Blueberry fruits contain exceptional amounts of anthocyanidins, flavonoids, organic acids, vitamin C, and antioxidants, and are famous for their high nutritional value and validated benefits for human health [3,4]. Generally, the timing of flowering varies according to plant species, plant age, intrinsic genetic components, various environmental factors, and even the cultivar and the corresponding cultivation techniques used [5,6,7]. For blueberry, flowering time is significantly associated with plant productivity and marketing time, and thus with the sale price of blueberry fruits [8,9]. To pursue substantial economic benefits, an increasing number of blueberry plants have been cultivated by forcing culture using very early to early ripening cultivars, such as ‘Misty’, ‘Emerald’, ‘O’Neal’, etc., under protected conditions [10,11] or in greenhouses and controlled rooms [12]. These conditions not only ensure safe overwintering but also earlier flowering and market time [13]. Recently, blueberry cultivation using a plant factory with artificial lighting (PFAL) system was introduced to improve fruit yields and quality, generally, in China, and the average blueberry fruit yield in greenhouse or open-field farms was approximately 5.27 tons per hectare [14,15]. The PFAL system is a modern and highly developed planting platform [16,17], inside which the growth parameters are always independent of seasonal changes and can be artificially manipulated according to plant growth dynamics [18,19]. However, the annual growth cycle of blueberries and especially the flowering event inside the PFAL system is significantly different from that of traditional open-field farms or greenhouses. Knowledge about flower bud initiation, differentiation, development, and blooming, as well as the possible regulatory mechanism associated with the biological flowering process of blueberry in PFAL systems is still very limited. Therefore, research into blueberry flower bud development and elucidation of the underlying mechanism that controls the flowering event in closed PFAL environments is urgently required.

Previous studies have confirmed that blueberry flower bud differentiation and development is a phytochrome-mediated short-day (SD) response, which usually occurs under an 8 h photoperiod, but temperature also has a profound effect on the photoperiodic induction of flower buds [20,21]. For instance, flower bud initiation in the lowbush blueberry (*Vaccinium angustifolium* Ait.) occurred under 11 or 13 h photoperiods with temperatures of either 10 or 21 °C, and flowers were produced under an 8 h photoperiod SD conditions for 2–4 weeks at constant 18 °C [22]. In rabbiteye blueberry (*Vaccinium ashei* Reade) [23], 6 weeks of inductive short days are required for normal flower bud formation. In addition, blueberry is a chilling-mediated flowering species that requires a certain number of chilling hours before flowering; fully chilled blueberry plants flower normally, whereas non-chilled plants might not flower [24]. Under natural conditions, most blueberry cultivars initiate flower buds in autumn and bloom the following spring [25]. In Shanghai, China, the blueberry buds begin to differentiate in July as soon as the harvest is finished, under a SD length rhythm. In this period, the subtropical hot and humid climate forces the new shoots to stop expanding and quickly cap off, and the process of flower bud differentiation lasts until late November or early December. The buds then undergo a shallow dormancy, begin to swell rapidly, and bloom once the chilling requirement has been satisfied. To reveal the genetic and physiological mechanisms of blueberry flowering events, numerous studies have been conducted in recent decades, and a large number of flowering-related genes have been successfully identified, such as *VcFT* [26,27], *SOC1*-like MADS-box [28], *VcRR2* [29], *SPL* [13], and *VcSVP* [30]. *FT* is a major integrator of signaling that stimulates the transition of meristem tissue into flower buds [31], is expressed mainly in leaves, and the resultant FT protein travels through the phloem to the shoot apical meristem, where it induces flower determination [32]. Constitutive expression of *VcFT* enables precocious flowering in transgenic blueberry plants, indicating that *VcFT* positively induces flower bud formation [26,27]; however, *VcFT* does not seem to be the only factor affecting chilling-mediated blueberry flowering [24]. Thus, the relationship between flowering and chilling levels needs further study to determine whether the SD length conditions could trigger bud differentiation in closed PFAL systems, and whether the differentiated buds could bloom successfully when chilling is introduced under SD conditions.

Transcriptome analysis is a powerful tool to identify differentially expressed genes (DEGs) and regulatory networks involved in complex biological processes. RNA-sequencing (RNA-Seq) technology has been successfully applied in blueberry to identify candidate genes or transcripts involving multiple specific traits, such as flowering [30,33], adventitious root formation [34], anthocyanin accumulation [35,36], flavonoid biosynthetic pathway [37], and fruit development [38,39]. Therefore, to better understand the process of flower bud development, the effects of SD length and chilling on flower bud initiation of blueberry in a PFAL system were surveyed in this study, and transcriptomic analysis was carried out to identify the key candidate flowering genes. This is the first report to reveal the molecular regulatory basis of flowering genes in blueberry using the PFAL system, which will contribute to the understanding of blueberry plant growth and flower development in plant factories with artificial lighting.

## 2. Results

### 2.1. Flower Bud Differentiation of ‘Misty’ in the PFAL System

**Figure 1:**
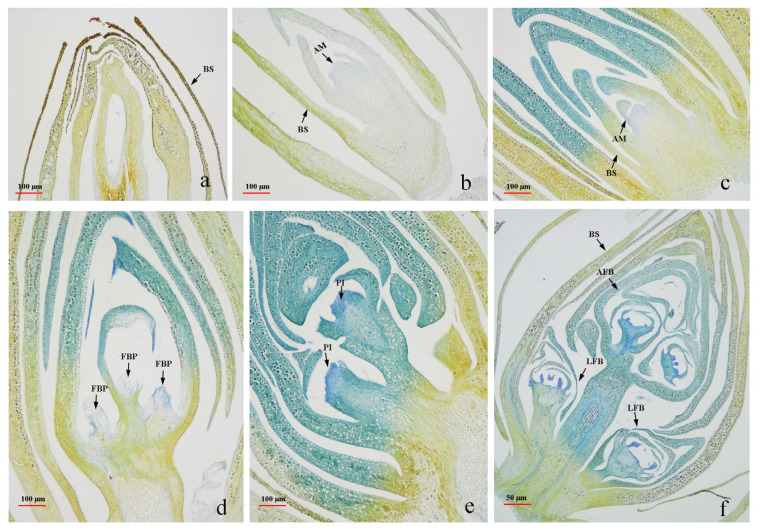
Observation of blueberry flower bud developmental process under short-day-length conditions in the PFAL system. Notes: subfigures (**a**–**f**) indicate the corresponding anatomical structure at sampled date of B1–B6, respectively. B1 indicates the date when the photoperiod was changed to an SD length, i.e., 8/16 h (marked as 0 d); B2 indicates 10 days after SD treatment; B3 indicates 20 days after SD treatment; B4 indicates 30 days after SD treatment; B5 indicates 50 days after SD treatment + 20 days after chilling (10 ± 2 °C, day/night) treatment; B6 indicates 60 days after SD treatment + 30 days after chilling treatment. BS, bud scale; AM, apical meristem; FBP, flower bud primordium; PI, pistil; AFB, apical flower bud; LFB, lateral flower bud; the bar indicates the scale.

To clarify the mechanism of blueberry flower bud differentiation in the PFAL system, we followed the bud developmental process of ‘Misty’ plants histologically. At the B1 stage, when the ‘Misty’ plants had been just switched from long-day (LD) length to SD length conditions, the apical meristem was small and closed (Figure 1a). At B2, 10 d after SD treatment, the bud scales began to loosen, and the apical meristems swelled and thickened to a hemispherical shape (Figure 1b). At B3, 20 d after SD treatment, the apical meristems further enlarged and became nearly elliptical (Figure 1c). At B4, 30 d after SD treatment, the flower bud primordium formed (Figure 1d). At B5, 50 d after SD treatment, when chilling was introduced for 20 d (SD + chilling treatment), the pistil primordium emerged (Figure 1e). At B6, after 60 d of SD treatment + 30 d of chilling, the differentiated flower buds, including apical and lateral flower buds, were observed (Figure 1f). The results suggest that blueberry flower buds can be initiated and differentiated in the PFAL system under SD length conditions combined with chilling treatment.

### 2.2. RNA-Sequencing and Identification of Differentially Expressed Genes

To understand the molecular basis of flowering bud differentiation of ‘Misty’ in PFAL under SD length conditions, six samples (B1, B2, B3, B4, B5, and B6) were subjected to total RNA extraction and transcriptome sequencing. After removing the adaptor and low-quality sequence, a total of 39.28 Gb of clean data were generated, containing 25.81–46.91 Mb of mapped reads, 25.45–30.17 Mb unique mapped reads, 35.59–57.01 Mb multiple mapped reads, and 20.31–24.11 Mb clean reads. The GC content ranged from 47.16 to 48.04%. The Q30 value for each library was >92.02% (Table 1).

### 2.3. Sequencing Annotation

All unique sequences were further annotated based on BLASTx searches against eight databases, i.e., clusters of orthologous genes (COG), GO, KEGG, KOG, NR, Pfam, Swiss-Prot, and evolutionary genealogy of genes: Non-supervised Orthologous Groups (eggNOG) databases. Using a cut-off *E*-value of 10^−5^, a high number of differentially expressed genes (DEGs) was identified, the number of annotated DEGs were shown detail in Table 2.

GO analysis was performed to classify the functions of the 21,287 predicted unigenes into three main categories: biological process (3468), cellular component (5480), and molecular function (12,339) (Figure S1). In the biological process category, unigenes involved in ‘metabolic process’, ‘cellular process’, and ‘single-organism process’ were dominant. In the cellular component category, a great number of unigenes were mainly categorized as ‘cell’, ‘cell part’, ‘membrane’, ‘organelle’, and ‘membrane part’. In the molecular function category, most of the unigenes were involved in ‘catalytic activity’ and ‘binding’ (Figure S1).

### 2.4. DEGs in Blueberry

**Figure 2:**
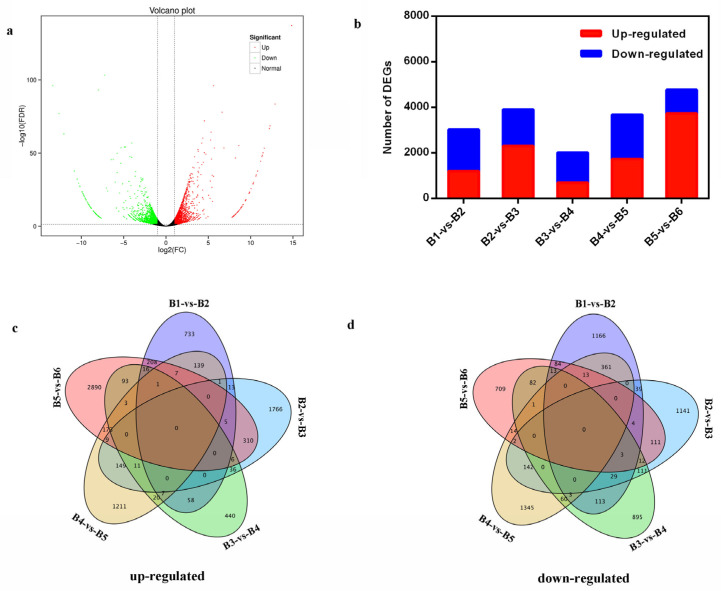
Statistics of differently expressed genes (DEGs). Note: (**a**) significantly up- or down-regulated unigenes using the −log10(*p*-value) and |log2FoldChange| ≥ 1 thresholds in B4-vs-B5; (**b**) graphical representation of overall DEGs in each library; (**c**,**d**) Venn diagram showing the number of up-regulated and down-regulated DEGs in each library.

Five DEGs libraries (B1-vs-B2, B2-vs-B3, B3-vs-B4, B4-vs-B5, and B5-vs-B6) were sequenced, and the FPKM values of all unigenes were calculated. Differences in gene expression were examined based on FDR ≤ 0.05 and |log2FoldChange| ≥ 1. DEGs were identified by pairwise comparison against the five libraries (Figure 2a). In total, 17,370 DEGs were detected, of which 9637 were up-regulated and 7733 were down-regulated (Figure 2b). A total of 3016 DEGs (1188 up-regulated and 1828 down-regulated), were identified in the B1-vs-B2 library; 3900 DEGs (2306 up-regulated and 1594 down-regulated) were detected in the B2-vs-B3 library; 2013 DEGs (691 up-regulated and 1322 down-regulated) were detected in the B3-vs-B4 library; 3672 DEGs (1731 up-regulated and 1941 down-regulated) were detected in the B4-vs-B5 library; and 4769 DEGs (3721 up-regulated and 1048 down-regulated) were detected in the B5-vs-B6 library (Figure 2b). However, there were no commonly up-regulated or down-regulated DEGs across all libraries, as illustrated in the Venn diagram (Figure 2c,d).

### 2.5. Flowering-Related DEGs of Blueberry in PFAL

**Figure 3:**
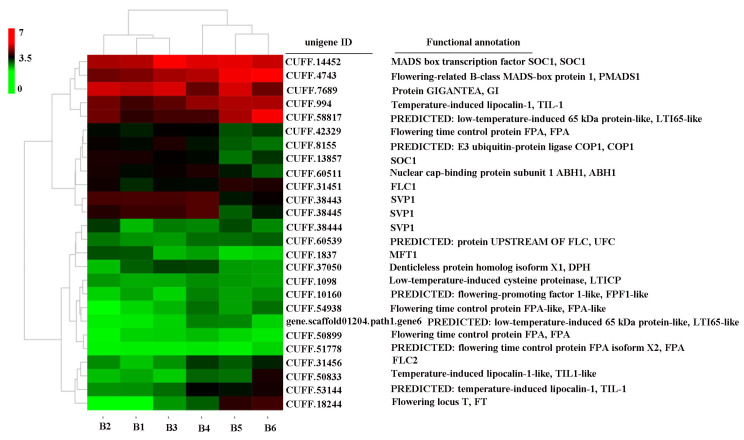
Heatmap of the expression of flowering-related DEGs identified by transcriptomic analysis of ‘Misty’ under short-day length in the PFAL system. Note: heatmap was created normalized FPKM value data using the Log_2_(1 + FPKM) formula.

Short-day length is one of the key environmental factors that induce blueberry apical buds to complete flower bud differentiation. In addition, differentiated flower buds require certain chilling or cooling hours conditions to bloom. Therefore, to identify the candidate DEGs involved in flowering and the chilling pathway, the distribution and functional annotation of unigenes was calculated based on the FPKM values and GO and KEGG enrichment with *p* = 0.05, and a total of 26 DEGs were identified, including 20 flowering-related and six low-temperature DEGs, and their gene expression patterns are illustrated in a heatmap (Figure 3).

### 2.6. Gene Expression Analysis

**Figure 4:**
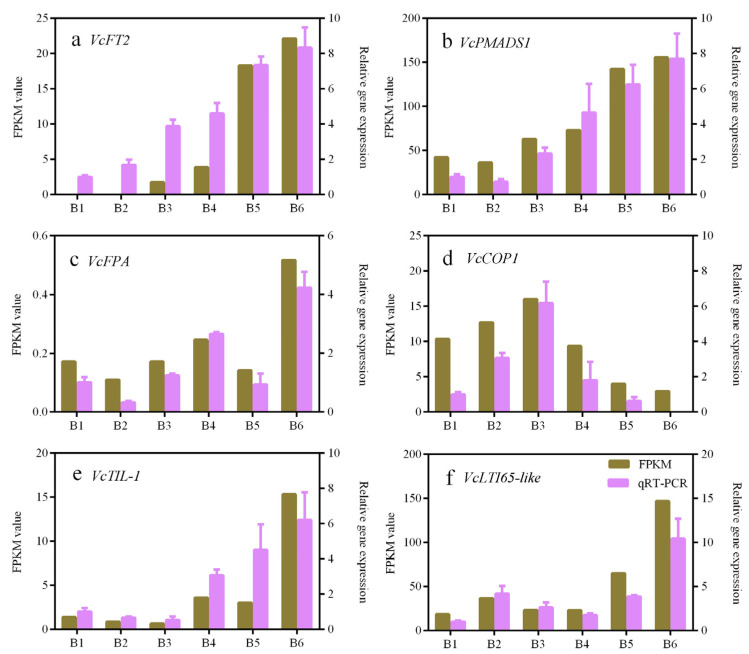
Expression patterns of *VcFT2* (**a**), *VcFMADS1* (**b**), *VcFPA* (**c**), *VcCOP1* (**d**), *VcTIL-1* (**e**), and *VcLTI65*-like (**f**) during floral bud differentiation of ‘Misty’ under short-day length in the PFAL system. Notes: Axis X indicates sampling time, e.g., B1, B2, B3, B4, B5, and B6; the left Y-axis indicates FPKM value; the right Y-axis indicates the relative gene expression levels verified by qRT-PCR; the bar indicates the standard error (n = 3).

To further analyze the DEG transcript trends along with SD duration and chilling treatment, six DEGs were further selected to analyze expression patterns in relation to sampling period based on the FPKM values: flowering locus T (*VcFT2*, unigene ID: CUFF.18244), flowering-related B-class *MADS-box* protein 1 (*VcFMADS1*, unigene ID: CUFF.4743), flowering time control protein *VcFPA* (unigene ID: CUFF.51778), E3 ubiquitin-protein ligase *VcCOP1* (unigene ID: CUFF.8155), temperature-induced lipocalin-1 (*VcTIL-1*, unigene ID: CUFF.50833), and low-temperature-induced 65 kDa protein-like (*VcLTI 65*-like, unigene ID: CUFF.58117) (Figure 4). The observation indicated that the expression patterns of *VcFT2*, *VcFMADS1*, and *VcFPA* were similar, and that the three genes were up-regulated gradually with SD treatment (Figure 4a–c). *VcCOP1* expression exhibited a trend opposite to those of *VcFT2*, *VcFMADS1*, and *VcFPA*. *VcCOP1* was expressed highly during the B1–B4 treatments, but down-regulated markedly in the B5 and B6 treatments (Figure 4d), suggesting that *VcCOP1* might play a negative regulatory role during blueberry flower bud differentiation in the PFAL system. The expression levels of *VcTIL-1* and *VcLTI 65*-like were relatively low; however, they were both up-regulated significantly during the chilling phase (B6 treatment) (Figure 4e,f), indicating that the two genes might be important for the chilling requirement stage (i.e., low-temperature-induced vernalization pathway). qRT-PCR analysis was performed to confirm the expression of the six DEGs, indicating a good reproducibility with the RNA-Seq data (Figure 4).

**Figure 5:**
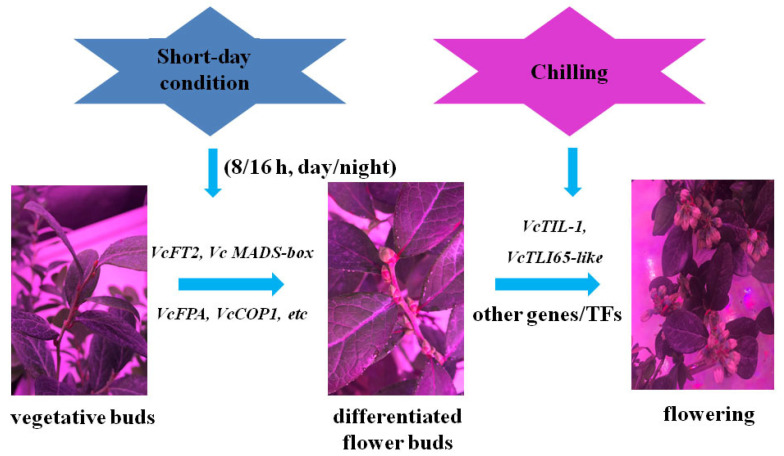
Assumed gene networks that regulate flower bud differentiation of blueberry under short-day length and chilling conditions in PFAL system.

Based on the reported chilling-mediated flowering pathway of blueberry reported in the literature [24] and the candidate DEGs identified in present study, a potential regulatory network that controls flower bud differentiation and thus flowering under SD conditions in PFAL system is derived. It is speculated that the SD length conditions would induce flower bud differentiation and trigger the expression of *VcFT2*, *VcFMADS1*, *VcFPA*, *VcCOP1*, and other flowering-related genes. When the temperature-associated genes or transcript factors, such as *VcTIL-1* and *VcLTI 65*-like genes, were stimulated for high expression following introduction of chilling, the blueberry plants eventually flowered (Figure 5).

### 2.7. Isolation and Characteristics Analysis of the VcFT2 Gene

**Figure 6:**
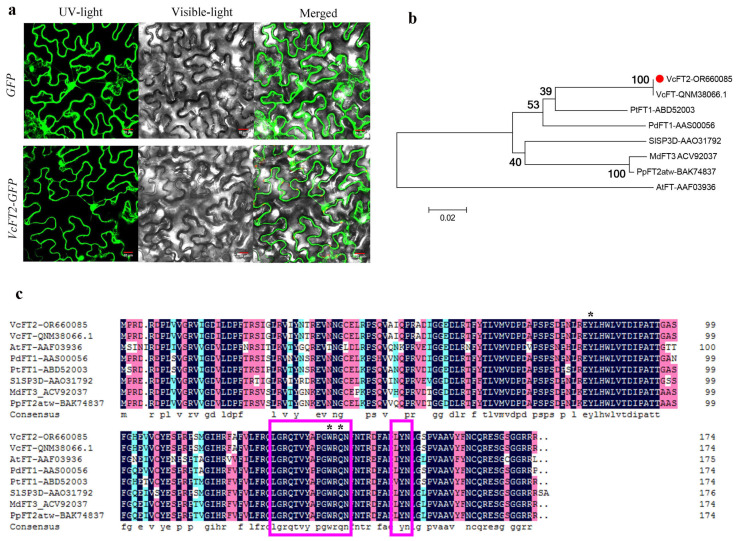
Subcellular localization of *VcFT2* in *Nicotiana benthamiana* leaves (**a**), phylogenetic analysis (**b**), and alignment of amino acid sequences of *VcFT2* and FT-like proteins from other plant species (**c**), including blueberry (*Vaccinium corymbosum*: VcFT, QNM38066), *Arabidopsis* (*Arabidopsis thaliana*: AtFT, AAF03936), tomato (*Solanum lycopersicum*: SlSP3D, AAO31792), poplar (*Populus deltoids*: PdFT1, AAS00056; *Poplar tremula*: PtFT1, ABD52003), apple (*Malus domestica*: MdFT3, ACV92037) and pear (*Pyrus pyrifolia*: PpFT2atw, BAK74837). Note: The bar in subfigure a was 20 μm. The derived protein of *VcFT2* was initially aligned to other known *FT* proteins using Clustal W. MEGA 6.0 was used to construct a phylogenetic tree using the neighbor-joining (NJ) method with 1000 bootstrap replications. Bootstrap percentages are shown at dendrogram branch points. The red circle in in subfigure b indicates the accession number of *VcFT2*. The unit for the scale bar in subfigure b displays branch lengths (0.02). Accession numbers are indicated at the right of the gene name. The asterisks indicate the conserved residues Tyr and Gln. The 14-amino-acid stretch (P-loop domain) and the LYN triad are marked with boxes.

The ORF of the *VcFT2* gene was 525 bp long and encoded a protein of 174 amino acids. For the subcellular localization assay, the coding region of *VcFT2* was fused to GFP to form the fusion gene *VcFT2::GFP* driven by *35S* promoter in Vector 1300. Both the fusion gene and GFP control plasmid were expressed transiently in *Nicotiana benthamiana* leaves. The fluorescence of *VcFT2::GFP* was observed simultaneously in the nucleus and cytoplasm (Figure 6a). The putative VcFT protein showed a high similarity (Figure 6b, 99.81%) to the known *Vaccinium corymbosum Flowering locus T* (MN708233.1), and when the nucleotide sequence was aligned against the NCBI database using BLASTn, only a single nucleotide difference was observed between the two *VcFT*s; therefore, the *VcFT* gene obtained in the present study was named *VcFT2* and deposited in GenBank with accession number OR660085. A comparison of the deduced amino acid sequences of *VcFT2* in GenBank indicated that *VcFT2* had high similarity with *FT*-like homologous of *Camellia sinensis* (AB741571.1, 85.90%) [40], *Actinidia chinensis* (KX611595.1, 84.38%) [41], *Fagus crenata* (AB775532.1, 82.29%) [42], *Hydrangea macrophylla* (MF374627.1, 82.29%), and *Betula luminifera* (JQ951966.1, 81.00%) [43], suggesting that the *VcFT2* obtained in the present study belonged to the FT-like superfamily. Subsequently, seven known *FT* genes, including *Vaccinium corymbosum* (VcFT, QNM38066) [27], *Arabidopsis thaliana* (AtFT, AAF03936) [44], *Solanum lycopersicum* (SlSP3D, AAO31792) [45], *Populus deltoids* (PdFT1, AAS00056) [46], *Poplar tremula* (PtFT1, ABD52003) [47], *Malus domestica* (MdFT3, ACV92037) [48], and *Pyrus pyrifolia* (PpFT2atw, BAK74837), were used to analyze the phylogenetic relationships among *VcFT2* and FT-like proteins, revealing that *VcFT2* had the closest kinship relationship with blueberry VcFT (Figure 6b). Multiple sequence alignments based on putative amino acid sequences indicated that *VcFT2* possessed typical features of the FT-like protein subfamily (Figure 6c). It not only contained the conserved amino acid residues Tyr (Y) and Gln (Q), but also a highly conserved 14-amino acid stretch (P-loop domain) and LYN motif (Figure 6c), suggesting that *VcFT2* is a putative ortholog of *FT*-like genes in blueberry.

**Figure 7:**
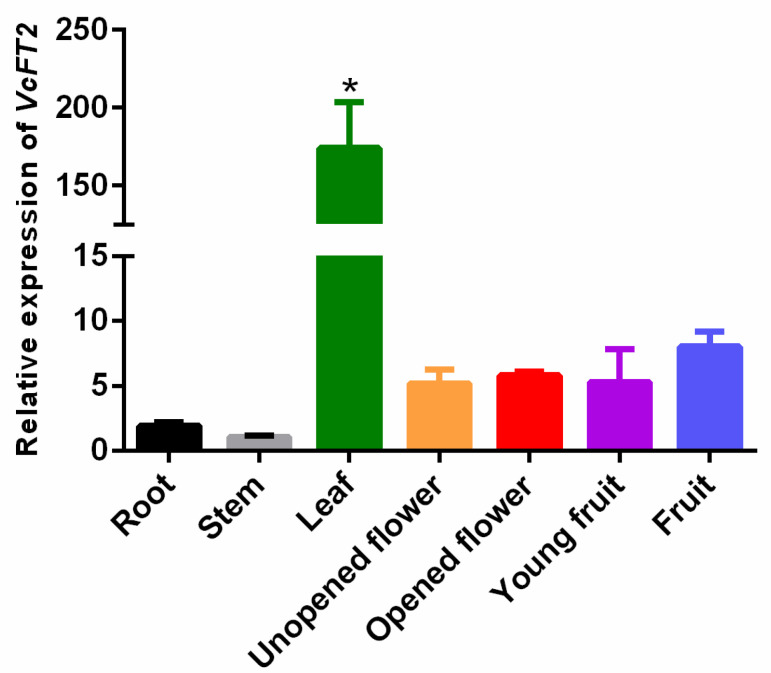
Tissue-specific expression of *VcFT2* in blueberry. Note: Roots, stems, and leaves were sampled at the same day when the new shoots stopped expanding. Flowers including unopened and opened flowers were collected when the blueberry plants began flowering. Young fruit was collected at the green fruit stage, and blue fruit indicated mature fruit. The bar indicates the standard error, * indicates significance at *p* ≤ 0.05.

To determine the expression patterns of *VcFT2* in blueberry, the tissue-specific expression of *VcFT2* was analyzed using quantitative real-time reverse transcription (qRT)-PCR. This indicated that *VcFT2* could be detected in all investigated tissues, with large differences in expression levels (Figure 7). *VcFT2* was expressed primarily in leaves, followed by higher expression in mature fruits, young fruits, opened flowers, and unopened flowers, with lower expression in root and stem tissues (Figure 7).

### 2.8. Ectopic Expression of VcFT2 Promotes Flowering in Arabidopsis

**Figure 8:**
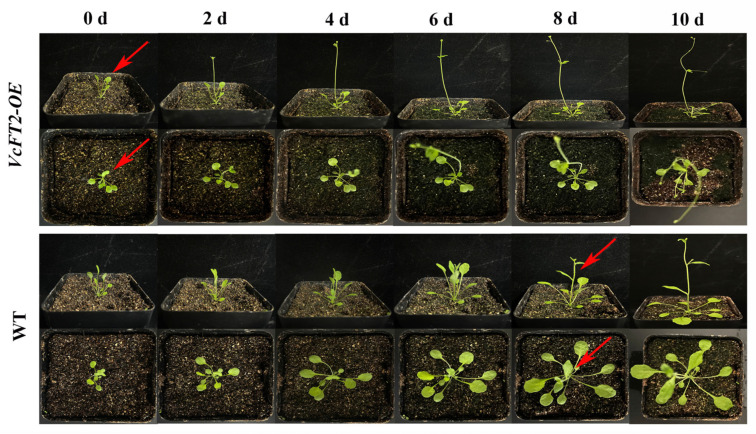
Effect of ectopic expression of *VcFT2* on phenotypic change in T3 transgenic *Arabidopsis thaliana*. Note: red arrow indicates the flower.

**Figure 9:**
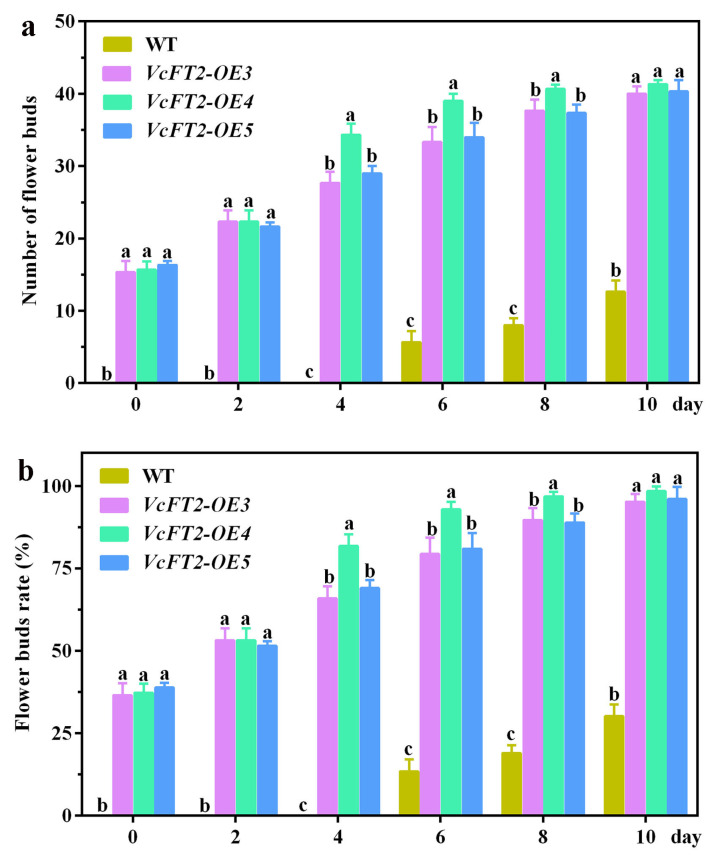
Statistics of flower bud number (**a**) and flower bud rate (**b**) for *VcFT2-OE* and WT plants. Notes: Different lowercase letters in column indicate significance at level of *p* ≤ 0.05.

**Figure 10:**
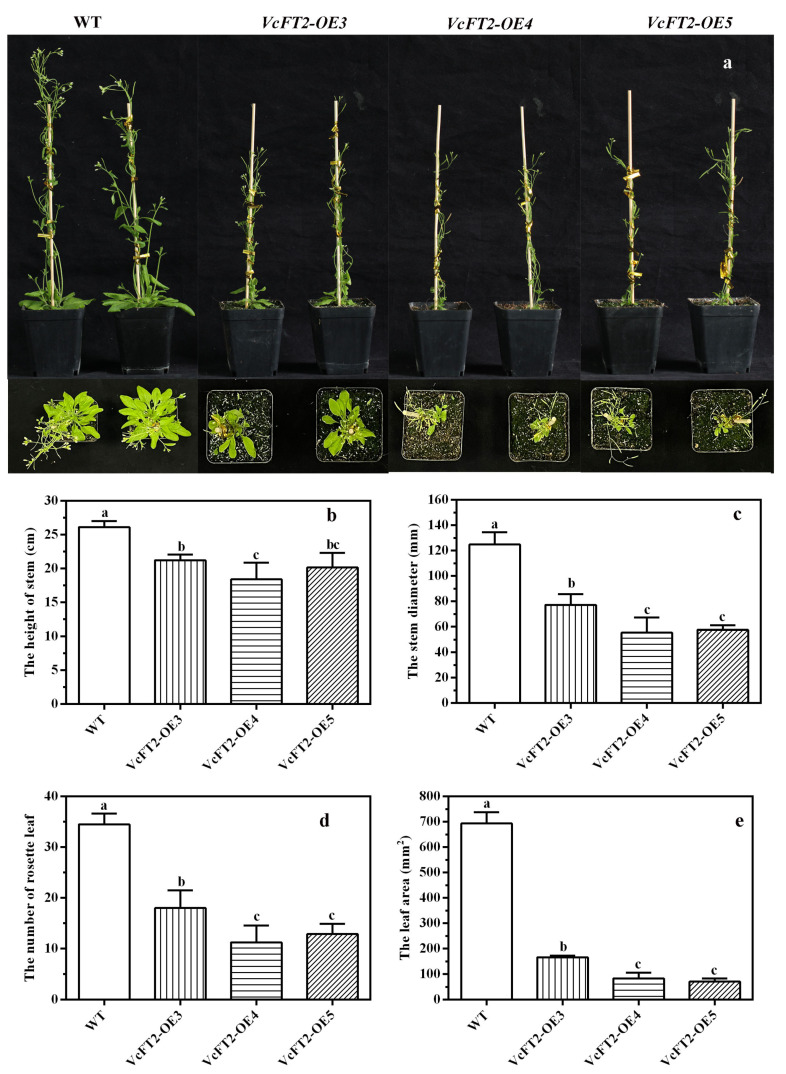
Phenotype observations of ectopically expressed *VcFT2-OE* and non-transgenic wild-type (WT) *Arabidopsis thaliana* 63 d after seed germination. Note: (**a**) growth phenotype transgenic *VcFT2-OE* and non-transgenic wild-type (WT) *Arabidopsis thaliana*; (**b**) stem height of *VcFT2-OE* and WT plants. (**c**) stem diameter of *VcFT2-OE* and WT plants; (**d**) rosette leaf number of *VcFT2-OE* and WT plants; (**e**) leaf area of *VcFT2-OE* and WT plants; Different lowercase letters in column indicate the significance at *p* ≤ 0.05.

To further investigate the function of *VcFT2*, transgenic *Arabidopsis* plants overexpressing *VcFT2* combined with *35S* promoter were generated, and three independent T3 lines, i.e., *VcFT2-OE3*, *VcFT2-OE4*, and *VcFT2-OE5*, were selected for flowering time analysis. The transgenic *VcFT2-OE* plants flowered at least 8 d earlier than the non-transgenic wild-type plants (Figure 8). Furthermore, both the number of flower buds and flower bud rate of the three lines *VcFT2*-*OE3*, *VcFT2-OE4*, and *VcFT2*-*OE5* were significantly higher than those of the wild-type plants (Figure 9a,b), suggesting that *VcFT2* in *Arabidopsis* might act as a flowering activator, thus leading to an earlier flowering phenotype. As for the transgenic lines, no significant differences in flower bud number and rate were observed among *VcFT2-OE3*, *VcFT2-OE4*, and *VcFT2-OE5* on day 0 to day 2, and day 10; however, the flower bud number and rate of *VcFT2-OE4* on day 4 to day 8 was significantly higher than those of *VcFT2-OE3* and *VcFT2-OE5* (Figure 9a,b). Vegetative growth of all transgenic lines of *VcFT2-OE Arabidopsis* was suppressed after approximately 63 d (Figure 10a). The plant height, stem diameter, rosette leaf number, and leaf area of *VcFT2-OE3*, *VcFT2-OE4*, and *VcFT2-OE5* were significantly lower than those of the non-transgenic wild-type plants (Figure 10b–e), indicating slower vegetative growth or dwarf traits of plants after *VcFT2* overexpression. As for the transgenic lines, the stem height of *VcFT2-OE3* was significantly higher than that of *VcFT2-OE4,* and the differences in stem height between *VcFT2-OE4* and *VcFT2-OE5* as well as that between *VcFT2-OE3* and *VcFT2-OE5* were not significant (Figure 10b). The stem diameter, rosette leaf number, and leaf area of *VcFT2-OE3* were significantly higher than those of *VcFT2-OE4* and *VcFT2-OE5*; however, there were no significant differences in stem diameter, rosette leaf number, and leaf area between *VcFT2-OE4* and *VcFT2-OE5* (Figure 10c–e).

## 3. Discussion

Bud differentiation in plant species such as blueberry is a complex physiological and biochemical process influenced by multiple factors including temperature, photoperiod, and day length. Blueberry is a photoperiod-sensitive berry shrub, and flower bud initiation appears to be promoted by exposure to short days, typically with a critical day length of less than 12 h [20,21,49]. Under controlled conditions, 8 weeks of 8, 10, or 12 h photoperiods will trigger and drive flower bud development in blueberries [19,22,23], and the flower bud number is positively associated with the length of exposure to short days [50]. Although highly advanced plant factory systems can provide a stable and high-efficiency growing environment for producing high-yield and high-quality blueberry fruits [14,15,51], the characteristics of the growth cycle of blueberry plants inside the PFAL system are poorly understood. In addition, it is uncertain whether the buds of blueberry plants can transition autonomously from the vegetative to the reproductive phase under controlled PFAL conditions. Sophisticated studies on temperature, day length, and photoperiod settings are needed to reveal the underlying flowering mechanisms in closed PFAL environments. In this study, an 8 h SD length (8/16 h day/night) was artificially set to induce flower bud differentiation using the low-chilled southern highbush ‘Misty’. Our observations of histoanatomical structures indicated that the apical bud of ‘Misty’ differentiated gradually along with the SD time course (Figure 1a–f), and when chilling (8/16 h day/night, 10 °C about 10–14 d) was introduced to satisfy the cold requirement levels of ‘Misty’, the differentiated buds visibly swelled. Moreover, when the indoor PFAL parameters were returned to 16/8 h (day/night) and 22 °C (the temperature was increased gradually), the differentiated buds burst and bloomed normally, a high fruit setting rate was recorded, and these blueberry fruits matured gradually (Figure S2). This demonstrates the possibility of inducing bud differentiation artificially in a PFAL system under certain SD photoperiod conditions.

Transcriptome analysis is a useful tool for identifying potential flowering pathway genes [52,53,54]. Using this approach, a large number of DEGs related to the flowering regulatory network have been identified in blueberry, such as the conserved flowering pathway genes *VcFT*, *VcFD*, *VcTFL1*, *VcLFY*, *VcAPP6*, and MADS-box genes; the photoperiod-related genes *VcCOL2* and *VcCOL5*; and some downstream genes of *VcFT* including *VcSOC1*, *VcAP1*, *VcCAL1*, and *VcFUL* [24,55]. These candidate DEGs have provided vital evidence for studying the mechanism of flowering in blueberry [56]. However, transcript information on blueberry flower bud development under SD length conditions in a PFAL system has not been reported. In the present study, comparative transcriptome analyses performed against different stages of floral bud in a PFAL system generated 20.31–24.11 Mb clean reads (Table 1) and numerous unigenes participating in blueberry floral bud development were screened out (Table 2, Figure 2 and Figure 3). After functional annotation, six key candidate DEGs involved in the flowering pathway were identified, namely *VcFT2*, *VcFMADS1*, *VcFPA*, *VcCOP1*, *VcTIL-1*, and *VcLTI 65*-like genes. These DEGs were significantly up-regulated or down-regulated along with the time course of SD length (Figure 4 and Figure 5), indicating their potential roles in the SD-mediated blueberry flowering pathways. These results were partly consistent with those reported by Song and Chen [24]. However, the functional characteristics and molecular mechanisms of the flowering DEGs identified in this study remain to be elucidated.

*FLOWERING LOCUS T* (*FT*) is a major flowering pathway integrator and a universal promoter of flowering, and functional analyses of *FT* and *FT*-like genes in numerous plant species have been reported [57,58]. *FT* protein has a highly conserved P-loop domain with a 14 amino acid (LGRQTVYAPGWRQN) motif and conserved YLH and LYN triads [59,60]. To verify *FT*-like genes in blueberry, in this study, a 525 bp *VcFT2* homolog was cloned from the flower bud of ‘Misty’ based on RNA-Seq data (Figure 6). Our results showed subcellular localization of *VcFT2* in the nucleus and cytoplasm (Figure 6a). The phylogenetic tree suggested that *VcFT2* clustered closely with *FT* or *FT*-like genes identified in the blueberry cultivar ‘Bluecrop’ and other species, and the sequence of *VcFT2* was highly conserved (Figure 6b,c). Alignment-based nucleotide sequences suggested that the CDs of *VcFT* and *VcFT2* only had a single nucleotide difference, possibly because the blueberry cultivar used to isolate *FT* was different, or because of the heterozygosity of the blueberry chromosome. Day length or photoperiod is perceived by leaves and induces a special signal called a florigen that moves through the phloem to the shoot apex [61,62,63]. Many *FT* or *FT*-like genes have been found to be highly expressed in leaves and are regulated by photoperiod, such as *FaFT1* in strawberries [64] and *GmFT2a* in soybeans [65]. Although *FT* is a mobile signal originating from leaves [66,67], a large number of *FT* or *FT*-like genes have been found mainly in reproductive tissues, such as *JcFT*, which is highly expressed in the flower buds of *Jatropha curcas* L. [68] and *GhFT1* expressed primarily in the stamens, sepals, petals, and carpels of *Gossypium hirsumtum* L. [69]. In the present study, *VcFT2* was highly expressed in leaves and secondarily in fruits and flowers (Figure 7). This is partly consistent with the findings in other species described above, indicating not only divergent expression patterns of *VcFT* homologs in blueberries, but also suggesting that *FT* genes might play multifaceted roles in plant development in addition to flowering time control [58,70]. In addition, ectopic overexpression of *VcFT2* in *Arabidopsis thaliana* L. resulted in an early flowering phenotype (Figure 8, Figure 9 and Figure 10), indicating that the *VcFT2* gene isolated in this study might act as a floral stimulator, that is, promote the transition from the vegetative to the reproductive phase. The results obtained in this study were consistent with those previously published by the Song research group [27,28,55]. However, the related functional genes or transcription factors (TFs) upstream and downstream of *VcFT2* remain unclear, and further study of *VcFT2* and its related flowering regulatory genes or TFs is required. Future studies should focus on mining and verifying the crosstalk between *VcFT2* and its upstream and downstream interacting genes. Furthermore, how *VcFT2* participates in the response of blueberry plants to photoperiod (SD) and temperature (chilling) conditions, and thus, the regulation of flowering, is unknown.

## 4. Materials and Methods

### 4.1. Plant Samples and Experimental Design

**Figure 11:**
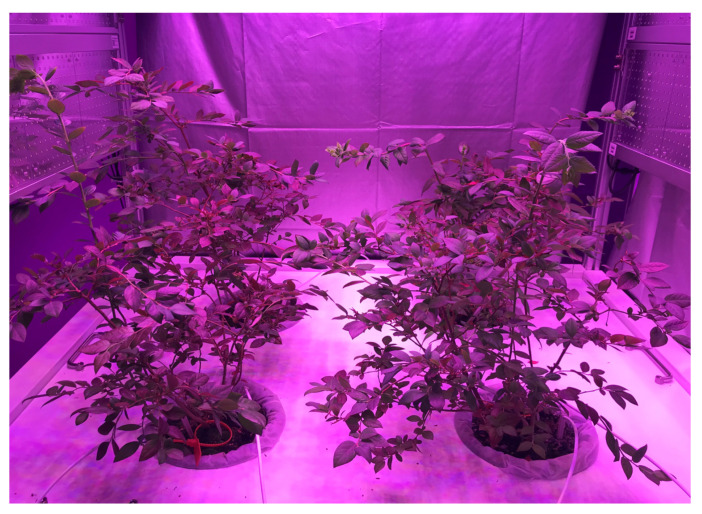
Blueberry plants grown under short-day-length conditions in the PFAL system.

Three-year-old southern highbush blueberry cultivar (*Vaccinium corymbosum* ‘Misty’) plants were used in the present study (Figure 11). In total, 24 ‘Misty’ plants were grown in room 1 of the PFAL system. All the plants were potted in the medium containing peatmoss/perlite/vermiculite (1:1:1, *v*/*v*/*v*) [14] and were irrigated daily using an automatic AEtrium-4 system (AEssence Grows, Santa Clara, CA, USA) with modified Hoagland nutrient solution (N:P:K = 1:1:1, pH = 5.0 ± 0.5, EC-value was 1500 ± 200 μS/cm). The plant height, stem diameter, shoot number, and shoot length of ‘Misty’ plants used in the study were 45.88 ± 12.13 cm, 10.91 ± 0.57 mm, 16.75 ± 3.89, and 24.86 ± 6.54 cm, respectively. Before treatment, the PFAL environmental parameters were set as follows: room temperature of 25 ± 2 °C/22 ± 2 °C (day/night), photoperiod of 16/8 h (day/night), CO_2_ concentration of 400 μmol mol^−1^, relative air humidity of 60–70%, and light intensity over blueberry canopy of 200 μmol m^−2^ s^−1^ [14]. Generally, in greenhouse or open-field farms, blueberry cultivars begin to differentiate flower buds under an SD length rhythm; however, the differentiated flower buds require a fixed quantity of chilling hours during winter endo-dormancy for vernalization [71], and over 80% of flower buds cannot bloom normally without a chilling period [24]. However, the characteristics flower bud formation and flowering events in PFAL systems are not clear. Therefore, in the present study, SD in combination with chilling treatments were designed to induce blueberry flower bud differentiation. The present study was carried out from March to May in 2020, when new shoots of ‘Misty’ stopped expanding. The photoperiod was changed to an SD length, i.e., 8/16 h (marked as 0 d, B1, collected on 3 March 2020), to induce flower bud differentiation, and the other growth parameters remained unchanged. Apical buds from the shoots were sampled every 10 d for one month; i.e., the buds were sampled at 10 d (B2, collected on 13 March 2020), 20 d (B3, collected on 23 March 2020), and 30 d (B4, collected on 2 April 2020). Subsequently, all SD-treated ‘Misty’ plants were transferred into room 3 of the PFAL for chilling at 10 ± 2 °C (day/night) under the same photoperiod, i.e., 8/16 h (day/night). Apical buds were sampled at 20 d (B5, collected on 22 April 2020) and 30 d (B6, collected on 2 May 2020) after the chilling treatment.

### 4.2. Observation of Blueberry Bud Differentiation in the PFAL System

To observe the process of blueberry flower bud formation and differentiation in the PFAL system, ten apical buds were randomly collected at B1, B2, B3, B4, B5, and B6, as described above. The samples were fixed using formaldehyde–acetic acid–alcohol (FAA) fixation solution, which was consisted by formaldehyde, acetic acid and 70% alcohol with the ratio of 5:5:90 (*v*/*v*/*v*). Before observation, all the sampled buds were softened for about 14 d within 4% ethylenediamine solution following the method of An et al. [34]. Subsequently, dehydration was carried out using a graded ethanol series. Vitrification was performed using dimethylbenzene before embedding the samples in paraffin. Finally, 10 μm-thick sections were cut using a rotary microtome (LEICA, RM2265), and photographs were captured under a light microscope (NIKON ECLIPSE E200).

### 4.3. Total RNA Extraction, RNA-Sequencing, and De Novo Transcriptome Assembly

Ten apical buds were collected at each sampling date (B1, B2, B3, B4, B5, and B6), total RNA was extracted using the Polysaccharide- and Polyphenolic-rich RNAprep Pure Plant Kit (TIANGEN, Beijing, China) according to the manufacturer’s protocol. The RNA integrity was validated using an Agilent 2100 Bioanalyzer (Agilent, Santa Clara, CA, USA). RNA-Seq libraries were constructed using a TruSeq Stranded mRNA LT Sample Prep Kit (Illumina, San Diego, CA, USA) and applied to an Illumina NovaSeq 6000 sequencing system guide for RNA-Seq analysis by Biomarker Technologies Corporation (Beijing, China). Due to limited space in the blueberry plant factory, a small amount of apical bud samples (10 buds at each sampling period) were collected; therefore, the RNA-Seq analysis was performed only once with no biological replicates.

### 4.4. Sequence Annotation and Identification of DEGs

Gene functions were annotated by aligning the genes with the nucleotide (NT) sequences of the National Center for Biotechnology Information (NCBI), non-redundant (NR) [72], Swiss-Prot [73], Pfam [74], Kyoto Encyclopedia of Genes and Genomes (KEGG) [75], Clusters of Orthologous Groups of proteins (COGs) [76], eggNOG [77], and Gene Ontology (GO) databases [78]. Gene expression levels were calculated using the fragments per kilobase per million reads (FPKM) method, which is the mostly used RNA-Seq analysis method because both sequencing depth and unigene length are considered by the method. Differential expression analysis was performed using the edgeR package [79]. Genes with fold change ≥ 2 and FDR ≤ 0.05 were identified as DEGs.

### 4.5. Cloning and Sequence Analysis of VcFT2

The open reading frame (ORF) region of *VcFT2* was amplified from the cDNA of ‘Misty’ flower bud using a conserved degenerate primer pair of *VcFT2*-F: 5′-ATGCCACGGGATAGGGATCC-3′; *VcFT2*-R 5′-GCGTCGTCGTCCTCCTGAG-3′. The *VcFT2*-F/R primer was designed based on the reference *FT* sequence (unigene ID: CUFF.18244) annotated using transcriptome data. The obtained sequence of *VcFT2* was analyzed using DNAman 6.0 (LynnonBiosoft, Quebec, QC, Canada) and aligned against the NCBI database using BLASTn (www.ncbi.nlm.nih.gov/blast.cgi, accessed on 10 June 2021). A neighbor-joining (NJ) phylogenetic tree of *VcFT2* and known *FT*s from other species was constructed using MEGA 6.0 software [80]. Bootstrap values were calculated from 1000 replications.

### 4.6. Subcellular Location

The CDs region of *VcFT2* without stop codon was amplified, then were fused with Green Fluorescent Protein (GFP) and inserted into pCAMBIA1300 vector. The transformation was carried out accordingly to the method described by Zuo et al. [81]. Agrobacterium (GV3101) transformed with the target vectors was suspended in the infiltration buffer with the value of OD_600_ was 0.6. Next, *Nicotiana benthamiana* leaves were injected. Infected plants were incubated for 48 h at 30 °C before observation under a NIKON C2-ER laser scanning confocal microscope (Nikon C2-ER, Nikon, Tokyo, Japan).

### 4.7. Arabidopsis Transformation

The CDS sequence of *VcFT2* was inserted after the *CaMV-35S* promoter in the pCAMBIA-1300-GFP vector to form the fusion vector *35S-VcFT2-GFP*. To further test the potential molecular biological function of *VcFT2*, the *Arabidopsis* Col-0 plants were transformed using *A. tumefaciens* containing the vector *35S-VcFT2-GFP* via the floral dip method [82]. Three T3 transgenic *Arabidopsis* lines (*VcFT2-OE3*, *VcFT2-OE4*, and *VcFT2-OE5*) possessed relatively high transcriptional levels of *VcFT2* and these transgenic lines were selected for subsequent experiments. The flowering time, flower bud number, and flower bud rate of *VcFT2* over-expressing plants and wild-type (WT) plants were recorded over a time course of 0–10 d after the *Arabidopsis* plants with 3–4 cotyledons were transplanted from the medium to soil composed by vermiculite and peat (2:1, *v*/*v*). For phenotype analyses, the stem height (cm), stem diameter (mm), rosette leaf number, and leaf area (mm^2^) of the 63-day *VcFT2* over-expressing plants and WT plants were measured. Each transgenic line or wild-type plant has three biological repeats and each biological repeat contains ten 63-day seedlings (i.e., the 63rd days after seeding).

### 4.8. Gene Expression Analysis by qRT-PCR

qRT-PCR analysis was performed according to the method of An et al. [34]. First-strand cDNA was synthesized using the PrimerScript^TM^ RT reagent Kit with gDNA Eraser (RR047, Takara, Japan), the qRT-PCR reaction was performed on a LightCycler 480 Real-Time PCR System (Roche, Basal, Switzerland), and the program was as follows: 95 °C for 30 s, then repeated for 40 cycles at 95 °C for 5 s and 60 °C for 20 s. *VcGAPDH* was used as the internal reference gene. Each qRT-PCR reaction was performed in triplicate and the expression level of each gene was calculated by the values of 2^−ΔΔCt^. The primer sequences for qRT-PCR analysis are listed in Supplementary Table S1.

### 4.9. Statistical Analysis

Data analysis was performed with one-way Analysis of Variance (ANOVA) using IBM SPSS Statistics 18 (IBM Corp., Armonk, NY, USA). Significant differences were compared by Duncan’s multiple range tests and *p* ≤ 0.05 was considered significant. Graphics were created using GraphPad Prism 6.0 (GraphPad Software Inc., San Diego, CA, USA).

## 5. Conclusions

Transcriptome analysis identified four flowering-related DEGs (*VcFT2*, *VcFPA*, *VcFMADS1*, and *VcCOP1*) and two low-temperature-induced DEGs (*VcTIL-1* and *VcLTI 65*-like) that play vital roles in the SD-mediated flowering pathway of blueberries. *VcFT2* was functionally conserved, with nuclear and cytoplasmic subcellular localization. Overexpression of *VcFT2* in *Arabidopsis* resulted in early flowering. These findings contribute to the comprehensive investigation of the SD-mediated flower bud differentiation mechanism of blueberries in PFAL systems. In future, the functional roles of these selected candidate DEGs should be elaborate to further reveal their regulatory mechanism that participates in SD or chilling–mediated flowering pathway of blueberry.

## Figures and Tables

**Table 1:** Summary of transcriptome of apical bud collected from blueberry cultivar *Vaccinium corymbosum* ‘Misty’ during flower bud differentiation in the PFAL system under short-day-length conditions.

Treatment		Total Reads	Mapped Reads	Uniq Mapped Reads	Multiple Mapped Reads	Clean Reads	GC Content (%)	Q30 (%)
Short-day length	B1	43,403,744	28,899,913 (66.58%)	28,531,813 (65.74%)	368,100 (0.85%)	21,707,872	47.22	93.39
	B2	40,616,476	25,810,648 (63.55%)	25,454,732 (62.67%)	355,916 (0.88%)	20,308,238	47.59	92.02
	B3	42,789,632	27,656,077 (64.63%)	27,300,033 (63.80%)	356,044 (0.83%)	21,394,816	47.16	94.45
	B4	42,026,350	27,049,542 (64.36%)	26,688,362 (63.50%)	361,180 (0.86%)	21,013,175	47.24	94.30
Short-day length + Chilling	B5	48,215,908	30,095,962 (62.42%)	29,644,912 (61.48%)	451,050 (0.94%)	24,107,954	48.04	94.57
	B6	46,094,392	30,743,814 (66.70%)	30,173,690 (65.46%)	570,124 (1.24%)	23,047,196	47.87	94.58

Note: B1–B6 indicate the sampled date; i.e., B1 indicates the date when the photoperiod was changed to an SD length, i.e., B1 indicates the date when the photoperiod was changed to an SD length, i.e., 8/16 h (marked as 0 d); B2 indicates 10 days after SD treatment; B3 indicates 20 days after SD treatment; B4 indicates 30 days after SD treatment; B5 indicates 50 days after SD treatment + 20 days after chilling (10 ± 2 °C, day/night) treatment; B6 indicates 60 days after SD treatment + 30 days after chilling treatment. GC indicates the GC content percentage of clean data, namely the percentage of clean data base G and C; Q30 indicates the base which quality value is greater than or equal to 30 percentage of clean data.

**Table 2:** Summary statistics of unigenes annotated by different databases.

Library	COG	GO	KEGG	KOG	NR	Pfam	Swiss-Prot	eggNOG
B1-vs-B2	759	1661	952	1301	2561	1850	1980	2431
B2-vs-B3	1014	2081	1200	1630	3205	2336	2502	3060
B3-vs-B4	431	926	505	759	1553	1066	1166	1465
B4-vs-B5	824	1809	1058	1506	2963	2088	2247	2835
B5-vs-B6	933	1982	1203	1800	3472	2271	2554	3190

Notes: Library (i.e., B1-vs-B2, B2-vs-B3, etc.) indicate pairwise between each two groups; the second column to the last represents the number of differentially expressed genes (DEGs) annotated by the functional databases separately.

## Data Availability

All raw data are deposited in the Sequence Read Archive (SRA) at NCBI under BioProject PRJNA1025873 with BioSample number SAMN37724062–SAMN37724067.

## Supplementary Materials

The following supporting information can be downloaded at: https://www.mdpi.com/article/10.3390/ijms25063197/s1.

## References

[1] An H., Meng J., Xu F., Jiang S., Wang X., Shi C., Zhou B., Luo J., Zhang X. (2019). Rooting ability of hardwood cuttings in highbush blueberry (*Vaccinium corymbosum* L.) using different indole-butyric acid concentrations. HortScience.

[2] Li Y., Pei J., Chen L., Sun H. (2021). China blueberry industry report 2020. J. Jilin Agric. Univ..

[3] Sivapragasam N., Neelakandan N., Rupasinghe H.P.V. (2023). Potential health benefits of fermented blueberry: A review of current scientific evidence. Trends Food Sci. Technol..

[4] Kim M., Na H., Kasai H., Kawai K., Li Y.S., Yang M. (2017). Comparison of Blueberry (*Vaccinium* spp.) and vitamin C via antioxidative and epigenetic effects in Human. J. Cancer Prev..

[5] Cho L.H., Yoon J., An G. (2017). The control of flowering time by environmental factors. Plant J..

[6] Reeves P.H., Coupland G. (2000). Response of plant development to environment: Control of flowering by daylength and temperature. Curr. Opin. Plant Biol..

[7] Tamada T. (1997). Flower-bud differentiation of highbush and rabbiteye blueberries. Acta Hortic..

[8] Lobos G.A., Hancock J.F. (2015). Breeding blueberries for a changing global environment: A review. Front. Plant Sci..

[9] Maust B.E., Williamson J.G., Darnell R.L. (2000). Carbohydrate reserve concentrations and flower bud density effects on vegetative and reproductive development in southern highbush blueberry. J. Am. Soc. Hortic. Sci..

[10] Santos B.M. (2013). Advances on protected culture of berry crops in Florida. J. Amer. Pomol. Soc..

[11] Ye Y.H., Aoki N., Konishi N., Patel N. (2005). Patterns of flower-bud differentiation, development, and growth habits of several blueberry (*Vaccinium* spp.) cultivar types grown in Japan and New Zealand. J. Jpn. Soc. Hort. Sci..

[12] Li Y., Sun H., Chen L. (2017). The blueberry industry of China: The past 10 years and the future. Acta Hortic..

[13] Feng X., Zhou B., Wu X., Wu H., Zhang S., Jiang Y., Wang Y., Zhang Y., Cao M., Guo B. (2023). Molecular characterization of SPL gene family during flower morphogenesis and regulation in blueberry. BMC Plant Biol..

[14] An H., Zhang J., Zhang L., Li S., Zhou B., Zhang X. (2023). Effects of nutrition and light quality on the growth of southern highbush blueberry (*Vaccinium corymbosum* L.) in an advanced plant factory with artificial lighting (PFAL). Horticulturae.

[15] Aung T., Muramatsu Y., Horiuchi N., Che J., Mochizuki Y., Ogiwara I. (2014). Plant growth and fruit quality of blueberry in a controlled room under artificial light. J. Jpn. Soc. Hort. Sci..

[16] Kozai T. (2013). Resource use efficiency of closed plant production system with artificial light: Concept, estimation and application to plant factory. Proc. Jpn. Acade. Ser. B..

[17] Kozai T. (2013). Plant factory in Japan-Current situation and perspectives. Chron. Hort..

[18] Kikuchi Y., Kanematsu Y., Yoshikawa N., Okubo T., Takagaki M. (2018). Environmental and resource use analysis of plant factories with energy technology options: A case study in Japan. J. Clean. Prod..

[19] Ohishi-Yamazaki M., Watanabe M., Nakanishi A., Che J., Horiuchi N., Ogiwara I. (2018). Shortening of the juvenile phase of the southern highbush blueberry (*Vaccinium corymbosum* L. interspecific hybrid) grown in controlled rooms under artificial light. Hort. J..

[20] Spann T.M., Williamson J.G., Darnell R.L. (2004). Photoperiod and temperature effects on growth and carbohydrate storage in southern highbush blueberry interspecific hubrid. J. Amer. Soc. Hort. Sci..

[21] Spann T.M., Williamson J.G., Darnell R.L. (2003). Photoperiodic effects on vegetative and reproductive growth of *Vaccinium darrowi* and *V. corymbosum* interspecific hybrids. HortScience.

[22] Hall I.V., Craig D.L., Aalders L.E. (1963). The effect of photoperiod on the growth and flowering of the highbush blueberry (*Vaccinium corymbosum* L.). Proc. Am. Soc. Hortic. Sci..

[23] Phatak S.C., Austin M.E. (1990). The effect of photoperiod on the growth and flowering of two rabbiteye blueberry cultivars. Appl. Agric. Res..

[24] Song G., Chen Q. (2018). Comparative transcriptome analysis of nonchilled, chilled and late-pink bud reveals flowering pathway genes involved in chilling-mediated flowering in blueberry. BMC Plant Biol..

[25] Omori M., Cheng C.C., Hsu F.C., Chen S.J., Yamane H., Tao R., Li K.T. (2022). Off-season flowering and expression of flowering-related genes during floral bud differentiation of rabbiteye blueberry in a subtropical climate. Sci. Hortic..

[26] Gao X., Walworth A.E., Mackie C., Song G. (2016). Overexpression of blueberry *FLOWERING LOCUS T* is associated with changes in the expression of phytohormone-related genes in blueberry plants. Hortic. Res..

[27] Song G., Walworth A., Zhao D., Jiang N., Hancock J.F. (2013). The *Vaccinium corymbosum FLOWERING LOCUS T*-like gene (*VcFT*): A flowering activator reverses photoperiodic and chilling requirements in blueberry. Plant Cell Rep..

[28] Song G., Walworth A., Zhao D., Hildebrandt B., Leasia M. (2013). Constitutive expression of the K-domain of a *Vaccinium corymbosum SOC1*-like (*VcSOC1-K*) MADS-box gene is sufficient to promote flowering in tobacco. Plant Cell Rep..

[29] Lin T., Walworth A., Zong X., Danial G.H., Tomaszewski E.M., Callow P., Han X., Zaharia L.I., Edger P.P., Zhong G. (2019). *VcRR2* regulates chilling-mediated flowering through expression of hormone genes in a transgenic blueberry mutant. Hortic. Res..

[30] Song G., Carter B.B., Zhong G.Y. (2023). Multiple transcriptome comparisons reveal the essential roles of *FLOERING LOCUS T* in floral initiation and SOC1 and SVP in floral activation in blueberry. Front. Genet..

[31] Kobayashi Y., Kaya H., Goto K., Iwabuchi M., Araki T. (1999). A pair of related gene with antagonistic roles in mediating flowering signals. Science.

[32] Yarur A., Soto E., Leon G., Almeida A.M. (2016). The sweet cherry (*Prunus avium*) *FLOWERING LOCUS T* gene is expressed during floral bud determination and can promote flowering in a winter-annual Arabidopsis accession. Plant Reprod..

[33] Song G., Gao X. (2014). Transcriptomic changes reveal gene networks responding to the overexpression of a blueberry *DWARF AND DELAYED FLOWERING 1* gene in transgenic blueberry plants. BMC Plant Biol..

[34] An H., Zhang J., Xu F., Jiang S., Zhang X. (2020). Transcriptomic profiling and discovery of key genes involved in adventitious root formation from green cuttings of highbush blueberry (*Vaccinium corymbosum* L.). BMC Plant Biol..

[35] Zhang J., Li S., An H., Zhang X., Zhou B. (2022). Integrated transcriptome and metabolome analysis reveals the anthocyanin biosynthesis mechanisms in blueberry (*Vaccinium corymbosum* L.) leaves under different light qualities. Front. Plant Sci..

[36] Han T., Wu W., Li W. (2021). Transcriptome analysis revealed the mechanism by which exogenous ABA increases anthocyanins in blueberry fruit during veraison. Front. Plant Sci..

[37] Li Y., Li H., Wang S., Li J., Bacha S.A.S., Xu G., Li J. (2023). Metabolomic and transcriptomic analyses of the flavonoid biosynthetic pathway in blueberry (*Vaccinium* spp.). Front. Plant Sci..

[38] Yang L., Liu L., Wang Z., Zong Y., Yu L., Li Y., Liao F., Chen M., Cai K., Guo W. (2021). Comparative anatomical and transcriptomic insights into *Vaccinium corymbosum* flower bud and fruit throughout development. BMC Plant Biol..

[39] Gupta V., Estrada A.D., Blakley I., Reid R., Patel K., Meyer M.D., Andersen S.U., Brown A.F., Lila M.A., Loraine A.E. (2015). RNA-Seq analysis and annotation of a draft blueberry genome assembly identifies candidate genes involved in fruit ripening, biosynthesis of bioactive compounds, and stage-specific alternative splicing. GigaScience.

[40] Li Q.S., Lin X.M., Qiao R.Y., Zheng X.Q., Lu J.L., Ye J.H., Liang Y.R. (2017). Effect of fluoride treatment on gene expression in tea plant (*Camellia sinensis*). Sci. Rep..

[41] Voogd C., Brian L.A., Wang T., Allan A.C., Varkonyi-Gasic E. (2017). Three *FT* and multiple *CEN* and *BFT* genes regulate maturity flowering, and vegetative phenology in kiwifruit. J. Exp. Bot..

[42] Miyazaki Y., Maruyama Y., Chiba Y., Kobayashi M.J., Joseph B., Shimizu K.K., Mochida K., Hiura T., Kon H., Satake A. (2014). Nitrogen as a key regulator of flowering in *Fagus crenata*: Understanding the physiological mechanism of masting by gene expression analysis. Ecol. Lett..

[43] Huang C., Song L., Tong Z., Cheng L. (2013). Cloning and expression analysis of *BlFTL* in *Betula luminifera*. J. Zhejiang A F Univ..

[44] Kardailsky I., ShuKLA V.K., Ahn J.H., Dagenais N., Christensen S.K., Nguyen J.T., Chory J., Harrison M.J., Weigel D. (1999). Activation tagging of the floral inducer *FT*. Sci..

[45] Carmel-Goren L., Liu Y.S., Lifschitz E., Zamir D. (2003). The *SELF-PRUNING* gene family in tomato. Plant Mol. Biol..

[46] Hsu C.Y., Liu Y., Luthe D.S., Yuceer C. (2006). Poplar *FT2* shortens the juvenile phase and promotes seasonal flowering. Plant Cell.

[47] Ziv D., Zviran T., Zezak O., Samach A., Irihimovitch V. (2014). Expression profiling of *FLOWERING LOCUS T-LIKE* gene in alternate bearing ‘Hass’ avovado trees suggests a role for *PaFT* in avocado flower induction. PLoS ONE.

[48] Kotoda N., Hayashi H., Suzuki M., Igarashi M., Hatsuyama Y., Kidou S., Igasaki T., Nishiguchi M., Yano K., Shimizu T. (2010). Molecular characterization of FLOWERING LOCUS T-Like gene of apple (*Malus* × *domestica* Borkh.). Plant Cell Physiol..

[49] Pescie M., Lovisolo M., Magistris A.D., Strik B., Lopez C. (2011). Flower bud iniation in southern highbush blueberry cv. O’Neal occurs twice per year in temperate to warm-temperate conditions. J. Appl. Hortic..

[50] Bañados M.P., Strik B. (2006). Manipulation of the annual growth cycle of blueberry using photoperiod. Acta Hortic..

[51] Hara H., Tarumi S., Hashimoto J., Sasaki Y., Tsuji N. (2013). Develop of small plant factory for enjoy cultivation of vegetables at home. Jpn. Soc. Sci. Des..

[52] Liu X., Yuan M., Dang S., Zhou J., Zhang Y. (2023). Comparative transcriptomic analysis of transcription factors and hormones during flower bud differentiation in ‘Red Globe’ grape under red-blue light. Sci. Rep..

[53] Wen Z., Guo W., Li J., Lin H., He C., Liu Y., Zhang Q., Liu W. (2017). Comparative transcriptmic analysis of vernalization- and cytokinin-induced floral transition in *Dendrobium nobile*. Sci. Rep..

[54] Yang M., Zhu L., Xu L., Pan C., Liu Y. (2014). Comparative transcriptomic analysis of the regulation of flowering in temperate and tropical lotus (*Nelumbo nucifera*). Ann. Appl. Biol..

[55] Walworth A.E., Chal B., Song G. (2016). Transcript profile of flowering regulatory genes in *VcFT*-overexpressing blueberry plants. PLoS ONE.

[56] da Silva M.N., Benevenuto J., Ferrao L.F.V., Munoz P.R. (2024). Genome-wide association study and transcriptome analysis reveal candidate genes for off-season flowering in blueberry. Sci. Hortic..

[57] Pin P.A., Benlloch R., Bonnet D., Wremerth-Weich E., Kraft T., Gielen J.J.L., Nisson O. (2010). An antagonistic pair of *FT* homologs mediates the control of flowering time in sugar beet. Science.

[58] Pin P.A., Nilsson O. (2012). The multifaceted roles of *FLOWERING LOCUS T* in plant development. Plant Cell Environ..

[59] Ahn J.H., Miller D., Winter V.J., Banfield M.J., Lee J.H., Yoo S.Y., Henz S.R., Brady R.L., Weigel D. (2006). A divergent external loop confers antagonistic activity on floral regulator FT and TFL1. EMBO J..

[60] Hanzawa Y., Money T., Bradley D. (2005). A single amino acid converts a repressor to an activator of flowering. Proc. Natl. Acad. Sci. USA.

[61] Lin M.K., Belanger H., Lee Y.J., Varkonyi-Gasic E., Taoka K., Miura E., Xoconostle-Cázares B., Gendler K., Jorgensen R.A., Phinney B. (2007). *FLOWERING LOCUS T* protein may act as the long-distance florigenic signal in the cucurbits. Plant Cell.

[62] Mathieu J., Warthmann N., Küttner F., Schmid M. (2007). Export of FT protein from phloem companion cells is sufficient for floral induction in *Arabidopsis*. Curr Biol..

[63] Faure S., Higgins J., Turner A., Laurie D.A. (2007). The *FLOWERING LOCUS T*-like gene family in barley (*Hordeum vulgare*). Genetics.

[64] Lei H., Guo X., Wang Y., Yao L., Wang S., Li T. (2015). Identification and characterization of *FaFT1*: A homolog of *FLOWERING LOCUS T* from strawberry. Adv. J. Food Sci. Technol..

[65] Sun H., Jia Z., Cao D., Jiang B., Wu C., Hou W., Liu Y., Fei Z., Zhao D., Han T. (2011). *GmFT2a*, a soybean homolog of *FLOWERING LOCUS T*, is involved in flowering transition and maintenance. PLoS ONE.

[66] Corbesier L., Vincent C., Jang S., Fornara F., Fan Q., Searle I., Giakountis A., Farrona S., Gissot L., Turnbull C. (2007). *FT* protein movement contributes to long-distance signaling in floral induction of *Arabidopsis*. Science.

[67] Tamaki S., Matsuo S., Wong H.L., Yokoi S., Shimamoto K. (2007). *Hd3a* protein is a mobile flowering signal in rice. Science.

[68] Li C., Luo L., Fu Q., Niu L., Xu Z.F. (2014). Isolation and functional characterization of *JcFT*, a *FLOWERING LOCUS T* (*FT*) homologous gene from the biofuel plant *Jatropha curcas*. BMC Plant Biol..

[69] Guo D., Li C., Dong R., Li X., Xiao X., Huang X. (2015). Molecular cloning and functional analysis of the *FLOWERING LOCUS T* (*FT*) homolog *GhFT1* from *Gossypium hirsutum*. J. Integr. Plant Biol..

[70] Lim C.W., Koh H., Lee S.C. (2023). The pepper phosphatidyl ethanolamine-binding proteins *CaMFT02* and *CaMFT03* have distinct roles in responses to drought and salt stress. Environ. Exp. Bot..

[71] Rowland L.J., Alkharouf N., Darwish O., Ogden E.L., Polashock J.J., Bassil N.V., Main D. (2012). Generation and analysis of blueberry transcriptome sequences from leaves, developing fruit and flower buds from cold acclimation through deacclimation. BMC Plant Biol..

[72] Deng Y.Y., Li J.Q., Wu S.F., Zhu T.P., Chen Y.W., He F.C. (2006). Integrated NR database in protein annotation system and its localization. Comput. Eng..

[73] Apweiler R. (2004). Uniprot: The universal protein knowledgebase. Nucleic Acids Res..

[74] Finn R.D., Bateman A., Clements J., Coggill P., Eberhardt R.Y., Eddy S.R., Heger A., Hetherington K., Holm L., Mistry J. (2013). Pfam: The protein families database. Nucleic Acids Res..

[75] Kanehisa M., Araki M., Goto S., Hattori M., Hirakawa M., Itoh M., Katayama T., Kawashima S., Okuda S., Tokimatsu T. (2008). KEGG for linking genomes to life and the environment. Nucleic Acids Res..

[76] Tatusov R.L., Galperin M.Y., Natale D.A., Koonin E.V. (2000). The COG database: A tool for genome-scale analysis of protein functions and evolution. Nucleic Acids Res..

[77] Huerta-Cepas J., Szklarczyk D., Forslund K., Cook H., Heller D., Walter M.C., Rattei T., Mende D.R., Sunagawa S., Kuhn M. (2016). EggNOG 4.5: A hierarchical orthology framework with improved functional annotations for eukaryotic, prokaryotic and viral sequences. Nucleic Acids Res..

[78] Ashburner M., Ball C.A., Blake J.A., Botstein D., Butler H., Cherry J.M., Davis A.P., Dolinski K., Dwight S.S., Eppig J.T. (2000). Gene ontology: Tool for the unification of biology. Nat Genet..

[79] Robinson M.D., McCarthy D.J., Smyth G.K. (2010). edgeR: A bioconductor package for differential expression analysis of digital gene expression data. Bioinformatics.

[80] Tamura K., Stecherm G., Peterson D., Filipskim A., Kumarm S. (2013). MEGA 6: Molecular evolutionary genetics analysis version 6.0. Mol. Biol. Evol..

[81] Zuo X., Wang S., Xiang W., Yang H., Tahir M.M., Zheng S., An N., Han M., Zhao C., Zhang D. (2021). Genome-wide identification of the 14-3-3 gene family and its participation in floral transition by interacting with TFL1/FT in apple. BMC Genom..

[82] Clough S.J., Bent A.F. (1998). Floral dip: A simplified method for *Agrobacterium*-mediated transformation of *Arabidopsis thaliana*. Plant J..

