# Supplementary material for: Transcriptome-Based Identification of Candidate Flowering-Associated Genes of Blueberry in a Plant Factory with Artificial Lighting (PFAL) under Short-Day-Length Conditions
Source: Int J Mol Sci. 2024 Mar 11;25(6):3197. doi: 10.3390/ijms25063197 (PMC10969908; doi:10.3390/ijms25063197)
Supplement: Supplementary file 1 [file ijms-25-03197-s001.zip › ijms-2907887-supplementary.pdf]

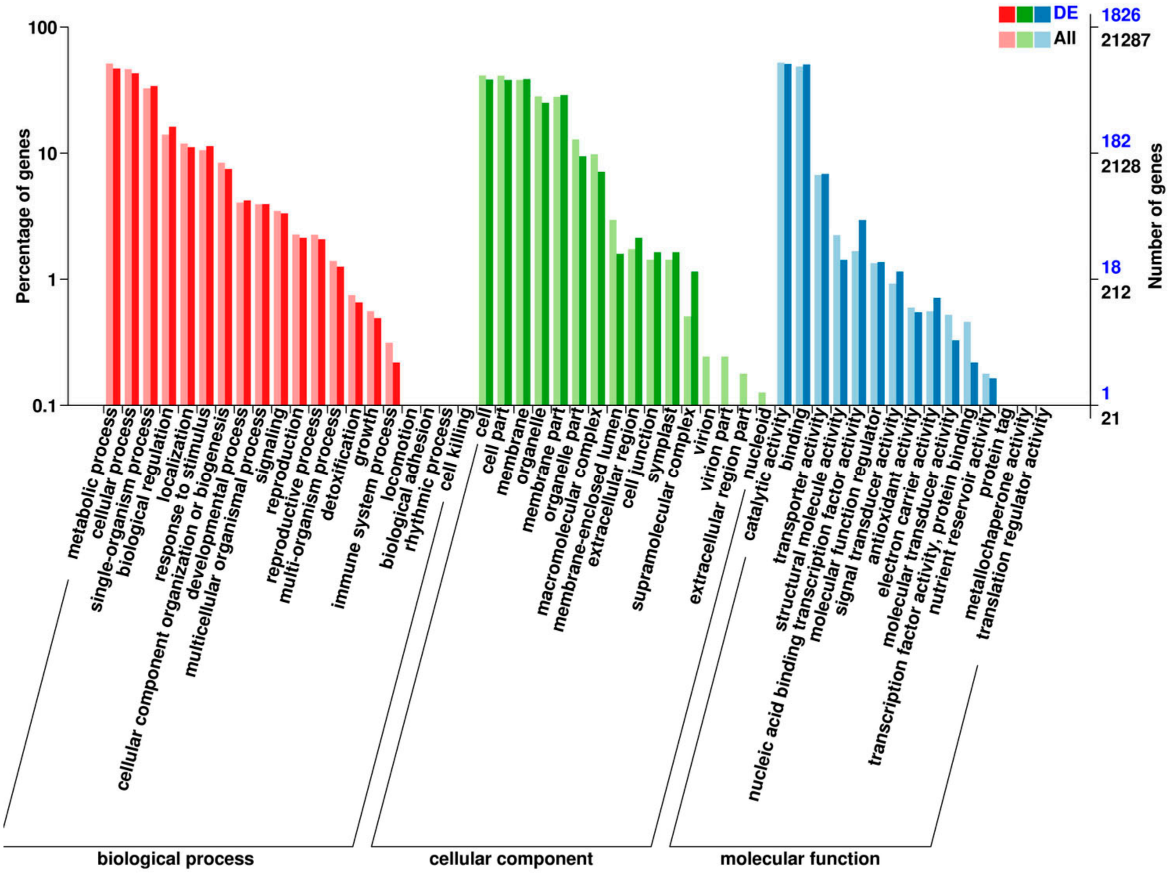

Figure S1. Functional annotation of blueberry transcriptome based on gene ontology categorization. A total of 21287 unigenes were categorized into three main categories: biological process (3468), cellular component (5480), and molecular function (12339). Note: The left y-axis indicates the percentage of a specific category of genes in the main category. The right y-axis indicates the number of genes in a category.

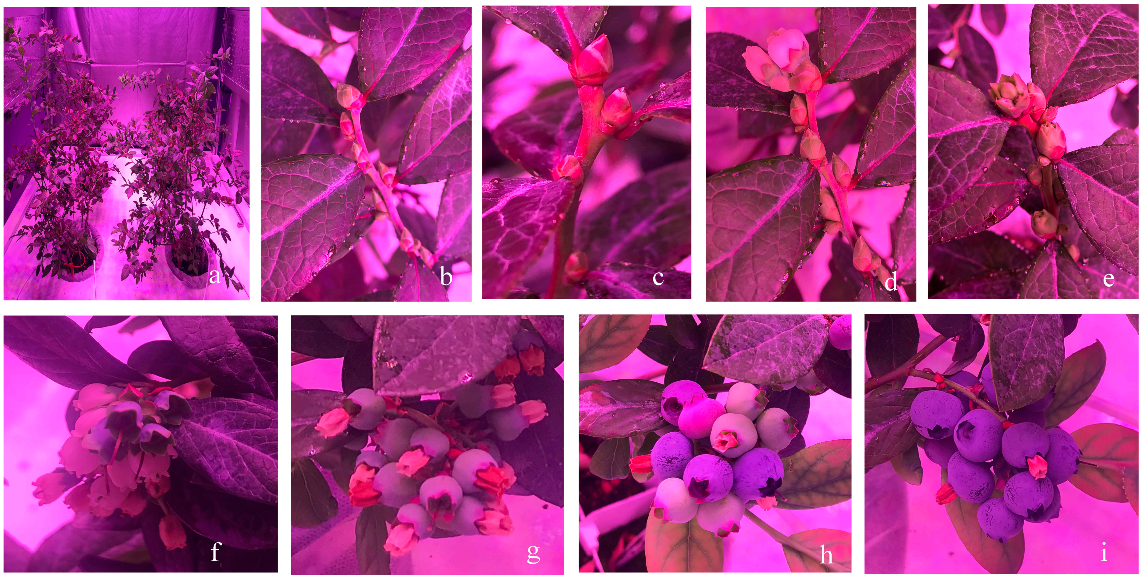

Figure S2. Flower bud differentiation and flowering and fruit development in 'Misty' in the PFAL system. Note: a, 'Misty' plants at vegetative growth stage; b, the differentiated buds under short-day (8/16h, day/night) condition; c, swollen buds after chilled treatment at 10 °C for about 10 d; d, the apical flower appeared when the

indoor PFAL was returned to long-day (16/8h, day/night) condition with 22°C for about two week; e, apical inflorescence appearance; f, fruit setting; g, green fruits began to enlarge; h, fruits began coloring; i, ripe fruits.

Table S1. Primers for qRT-PCR analysis used in this study.

Primers	Sequence (5′ to 3′)	Primers	Sequence (5′ to 3′)
VcFT2_Fwd	CCAAGTGACCCCAACCTAAG	*VcFT2_Rev*	CCACCTCATGTCCAAAGCTAG
VcPMADS1_Fwd	ACTACGACTACGACTACCCAG	*VcPMADS1_Rev*	ATGTTCCTGATCAAGCGAGG
VcFPA_Fwd	ATAGGGACACAACGAGCTTG	*VcFPA_Rev*	GGCTCTCCATCTCCTTCAAATG
VcCOP1_Fwd	GTTACATGTGCATTGCCACG	*VcCOP1_Rev*	AGTTCCATGTCTTGCTATCTGAC
VcTIL-1_Fwd	ATCGCCTCATTCCCATCAAG	*VcTIL-1_Rev*	TTTAGGACATGGACAGTGCC
VcLTI65-like_Fwd	CCGTCCCAATGTATGAGTATCC	*VcLTI65-like_Rev*	TTGTGGGTCTTGGTACTTGG
VcGAPDH_Fwd	ACTACCATCCACTCTATCACCG	*VcGAPDH_Rev*	AACACCTTACCAACAGCCTTG
